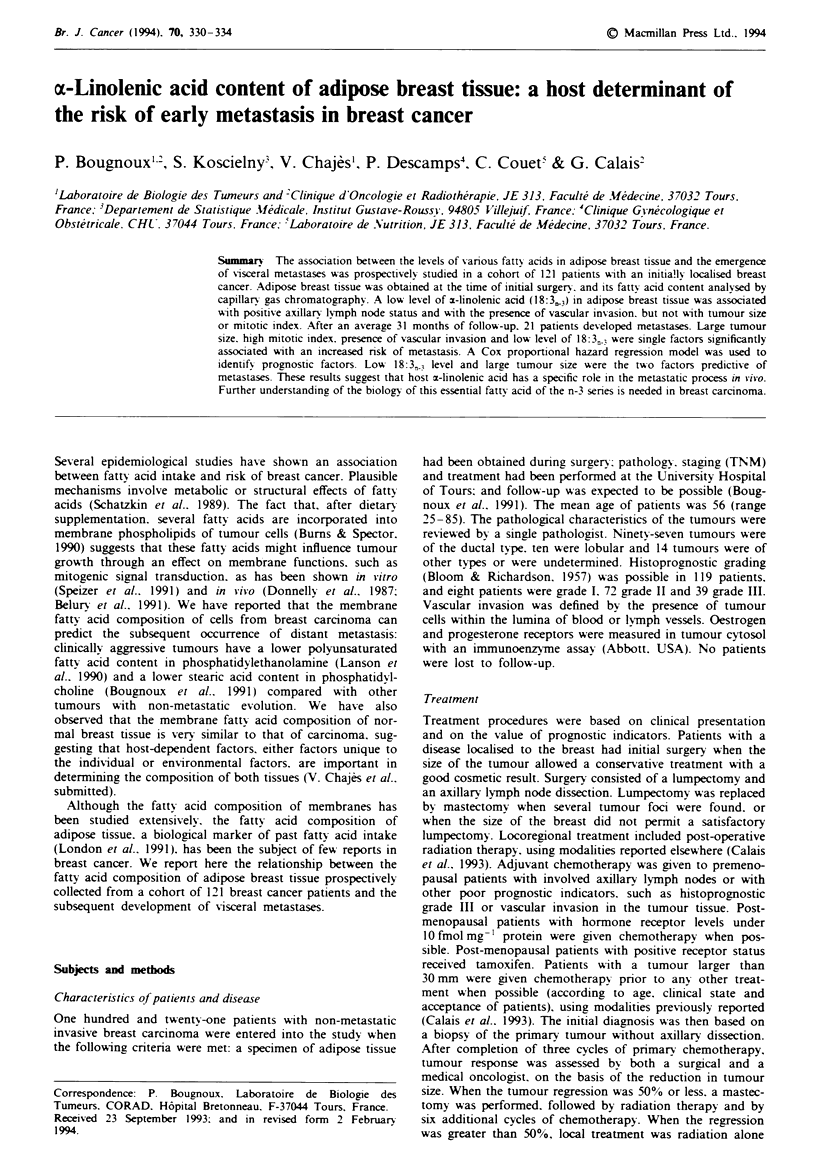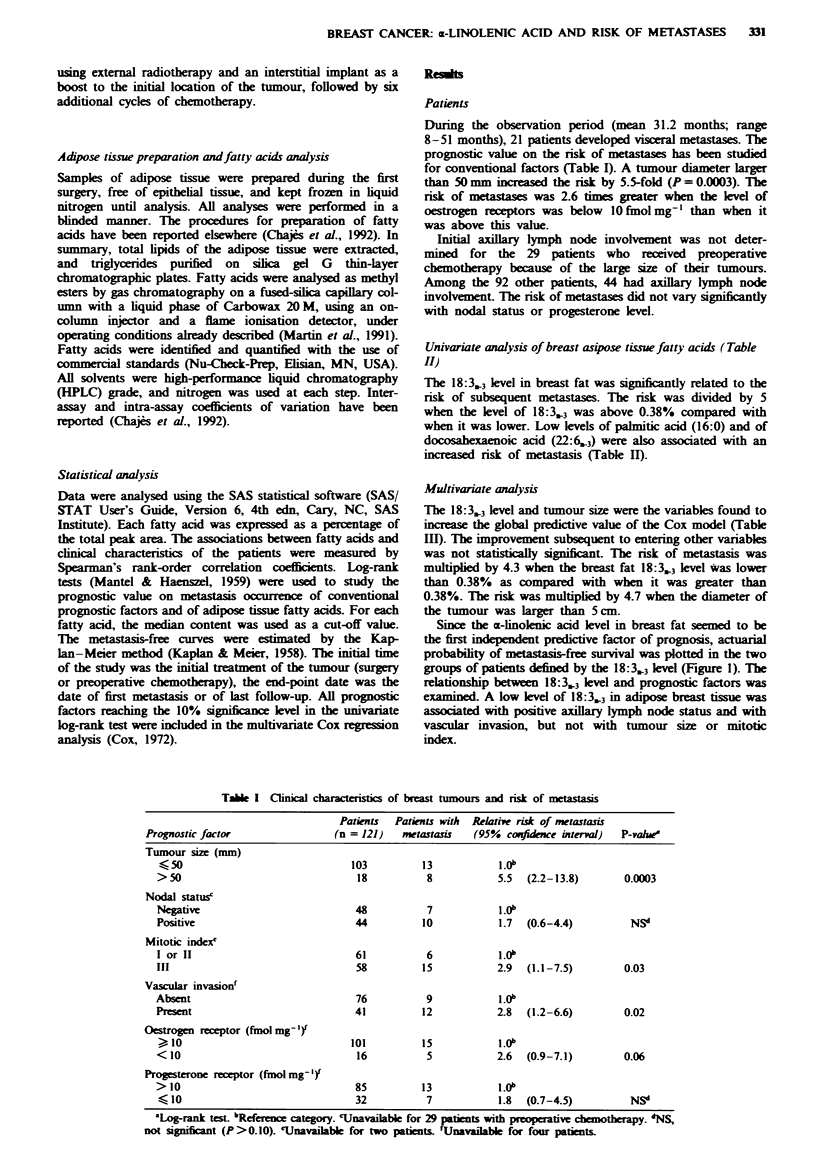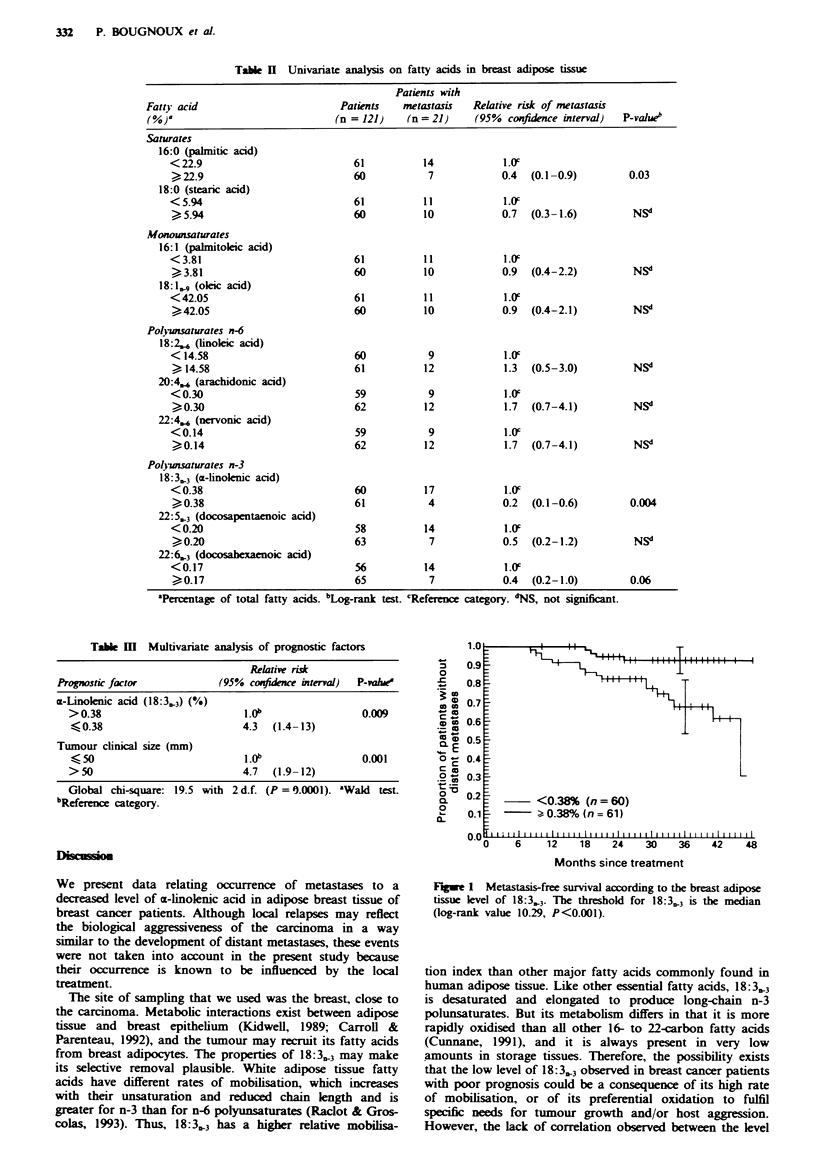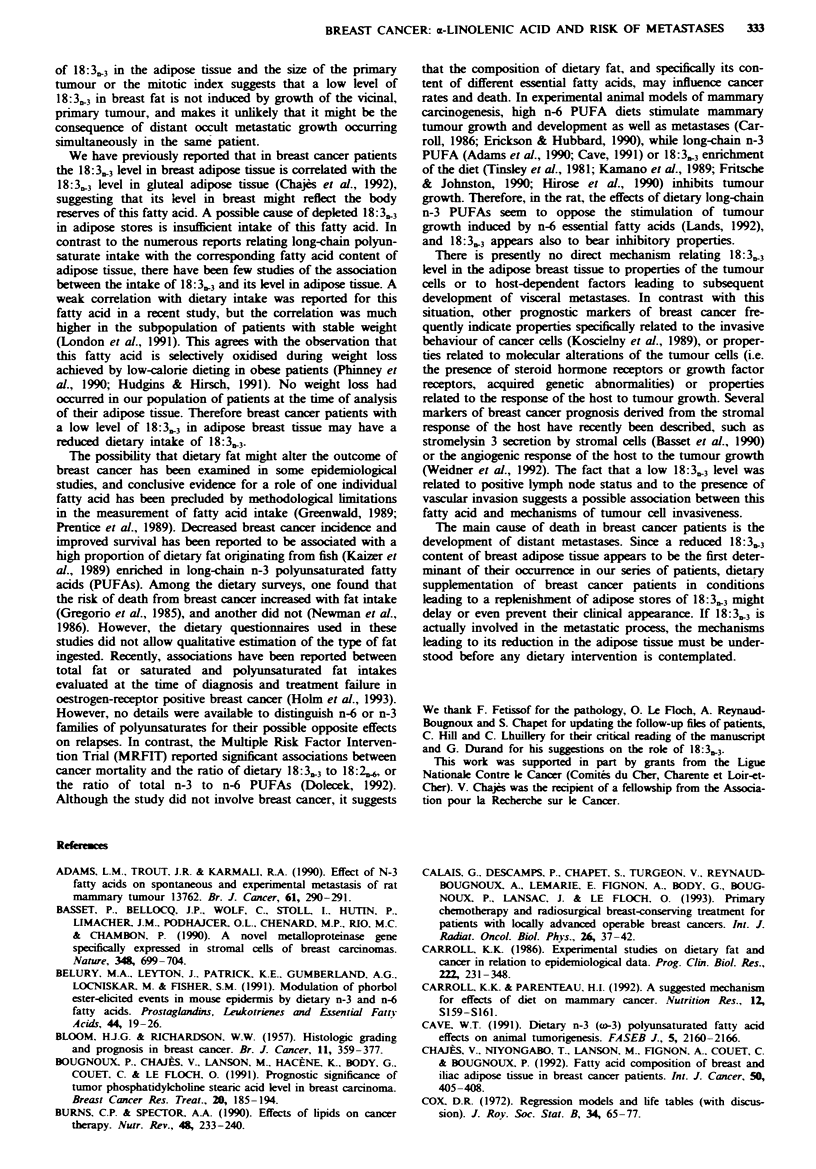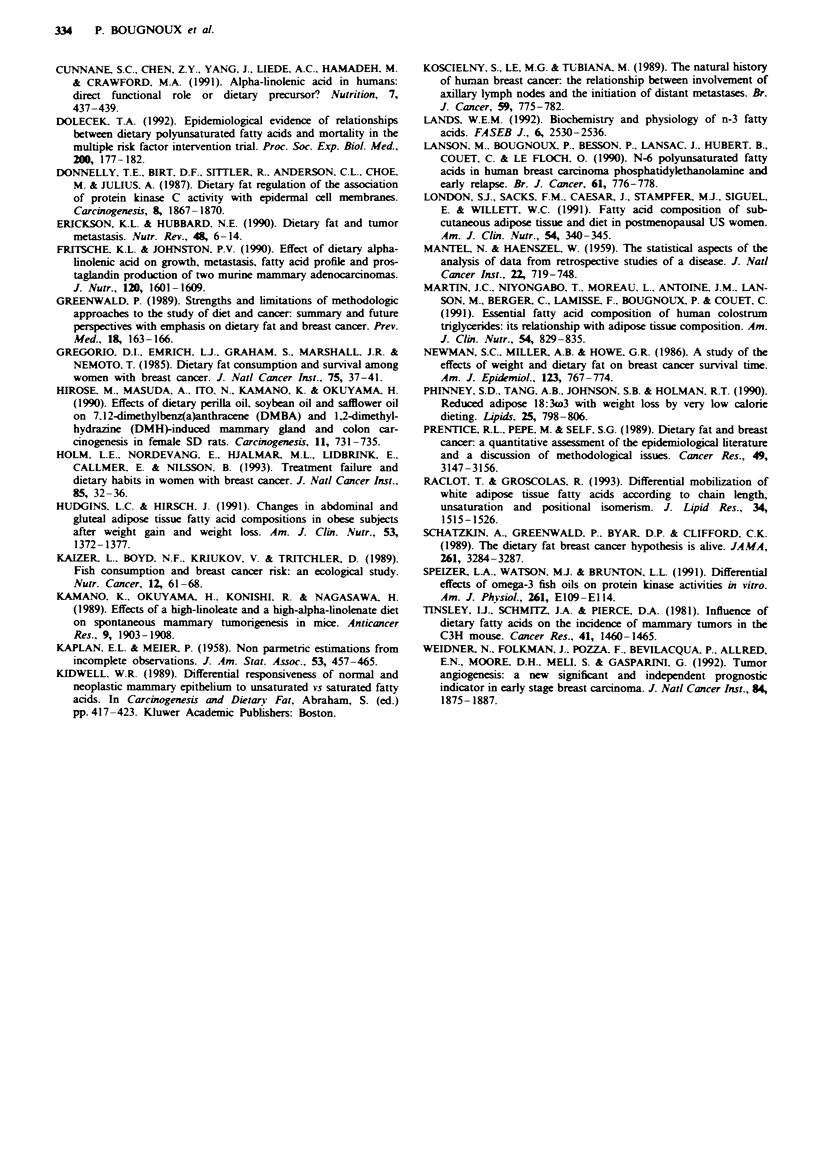# alpha-Linolenic acid content of adipose breast tissue: a host determinant of the risk of early metastasis in breast cancer.

**DOI:** 10.1038/bjc.1994.302

**Published:** 1994-08

**Authors:** P. Bougnoux, S. Koscielny, V. Chajès, P. Descamps, C. Couet, G. Calais

**Affiliations:** Laboratoire de Biologie des Tumeurs, Tours, France.

## Abstract

The association between the levels of various fatty acids in adipose breast tissue and the emergence of visceral metastases was prospectively studied in a cohort of 121 patients with an initially localised breast cancer. Adipose breast tissue was obtained at the time of initial surgery, and its fatty acid content analysed by capillary gas chromatography. A low level of alpha-linolenic acid (18:3n-3) in adipose breast tissue was associated with positive axillary lymph node status and with the presence of vascular invasion, but not with tumour size or mitotic index. After an average 31 months of follow-up, 21 patients developed metastases. Large tumour size, high mitotic index, presence of vascular invasion and low level of 18:3n-3 were single factors significantly associated with an increased risk of metastasis. A Cox proportional hazard regression model was used to identify prognostic factors. Low 18:3n-3 level and large tumour size were the two factors predictive of metastases. These results suggest that host alpha-linolenic acid has a specific role in the metastatic process in vivo. Further understanding of the biology of this essential fatty acid of the n-3 series is needed in breast carcinoma.


Br. J. Cancer (1994), 70, 330–334 © Macmillan Press Ltd., 1994

### α-Linolenic acid content of adipose breast tissue: a host determinant of the risk of early metastasis in breast cancer

P. Bougnoux[1,2], S. Koscielny[3], V. Chajès[1], P. Descamps[4], C. Couet[5] & G. Calais[2]

[1]Laboratoire de Biologie des Tumeurs and [2]Clinique d'Oncologie et Radiothérapie, JE 313, Faculté de Médecine, 37032 Tours, France; [3]Departement de Statistique Médicale, Institut Gustave-Roussy, 94805 Villejuif, France; [4]Clinique Gynécologique et Obstétricale, CHU, 37044 Tours, France; [5]Laboratoire de Nutrition, JE 313, Faculté de Médecine, 37032 Tours, France.

**Summary** The association between the levels of various fatty acids in adipose breast tissue and the emergence of visceral metastases was prospectively studied in a cohort of 121 patients with an initially localised breast cancer. Adipose breast tissue was obtained at the time of initial surgery, and its fatty acid content analysed by capillary gas chromatography. A low level of α-linolenic acid ($18{:}3_{n-3}$) in adipose breast tissue was associated with positive axillary lymph node status and with the presence of vascular invasion, but not with tumour size or mitotic index. After an average 31 months of follow-up, 21 patients developed metastases. Large tumour size, high mitotic index, presence of vascular invasion and low level of $18{:}3_{n-3}$ were single factors significantly associated with an increased risk of metastasis. A Cox proportional hazard regression model was used to identify prognostic factors. Low $18{:}3_{n-3}$ level and large tumour size were the two factors predictive of metastases. These results suggest that host α-linolenic acid has a specific role in the metastatic process *in vivo*. Further understanding of the biology of this essential fatty acid of the n-3 series is needed in breast carcinoma.

Several epidemiological studies have shown an association between fatty acid intake and risk of breast cancer. Plausible mechanisms involve metabolic or structural effects of fatty acids (Schatzkin *et al*., 1989). The fact that, after dietary supplementation, several fatty acids are incorporated into membrane phospholipids of tumour cells (Burns & Spector, 1990) suggests that these fatty acids might influence tumour growth through an effect on membrane functions, such as mitogenic signal transduction, as has been shown *in vitro* (Speizer *et al*., 1991) and *in vivo* (Donnelly *et al*., 1987; Belury *et al*., 1991). We have reported that the membrane fatty acid composition of cells from breast carcinoma can predict the subsequent occurrence of distant metastasis: clinically aggressive tumours have a lower polyunsaturated fatty acid content in phosphatidylethanolamine (Lanson *et al*., 1990) and a lower stearic acid content in phosphatidylcholine (Bougnoux *et al*., 1991) compared with other tumours with non-metastatic evolution. We have also observed that the membrane fatty acid composition of normal breast tissue is very similar to that of carcinoma, suggesting that host-dependent factors, either factors unique to the individual or environmental factors, are important in determining the composition of both tissues (V. Chajès *et al*., submitted).

Although the fatty acid composition of membranes has been studied extensively, the fatty acid composition of adipose tissue, a biological marker of past fatty acid intake (London *et al*., 1991), has been the subject of few reports in breast cancer. We report here the relationship between the fatty acid composition of adipose breast tissue prospectively collected from a cohort of 121 breast cancer patients and the subsequent development of visceral metastases.

### Subjects and methods

### *Characteristics of patients and disease*

One hundred and twenty-one patients with non-metastatic invasive breast carcinoma were entered into the study when the following criteria were met: a specimen of adipose tissue had been obtained during surgery; pathology, staging (TNM) and treatment had been performed at the University Hospital of Tours; and follow-up was expected to be possible (Bougnoux *et al*., 1991). The mean age of patients was 56 (range 25–85). The pathological characteristics of the tumours were reviewed by a single pathologist. Ninety-seven tumours were of the ductal type, ten were lobular and 14 tumours were of other types or were undetermined. Histoprognostic grading (Bloom & Richardson, 1957) was possible in 119 patients, and eight patients were grade I, 72 grade II and 39 grade III. Vascular invasion was defined by the presence of tumour cells within the lumina of blood or lymph vessels. Oestrogen and progesterone receptors were measured in tumour cytosol with an immunoenzyme assay (Abbott, USA). No patients were lost to follow-up.

### *Treatment*

Treatment procedures were based on clinical presentation and on the value of prognostic indicators. Patients with a disease localised to the breast had initial surgery when the size of the tumour allowed a conservative treatment with a good cosmetic result. Surgery consisted of a lumpectomy and an axillary lymph node dissection. Lumpectomy was replaced by mastectomy when several tumour foci were found, or when the size of the breast did not permit a satisfactory lumpectomy. Locoregional treatment included post-operative radiation therapy, using modalities reported elsewhere (Calais *et al*., 1993). Adjuvant chemotherapy was given to premenopausal patients with involved axillary lymph nodes or with other poor prognostic indicators, such as histoprognostic grade III or vascular invasion in the tumour tissue. Post-menopausal patients with hormone receptor levels under 10 fmol $mg^{-1}$ protein were given chemotherapy when possible. Post-menopausal patients with positive receptor status received tamoxifen. Patients with a tumour larger than 30 mm were given chemotherapy prior to any other treatment when possible (according to age, clinical state and acceptance of patients), using modalities previously reported (Calais *et al*., 1993). The initial diagnosis was then based on a biopsy of the primary tumour without axillary dissection. After completion of three cycles of primary chemotherapy, tumour response was assessed by both a surgical and a medical oncologist, on the basis of the reduction in tumour size. When the tumour regression was 50% or less, a mastectomy was performed, followed by radiation therapy and by six additional cycles of chemotherapy. When the regression was greater than 50%, local treatment was radiation alone

Correspondence: P. Bougnoux, Laboratoire de Biologie des Tumeurs, CORAD, Hôpital Bretonneau, F-37044 Tours, France.
Received 23 September 1993; and in revised form 2 February 1994.

using external radiotherapy and an interstitial implant as a boost to the initial location of the tumour, followed by six additional cycles of chemotherapy.

### *Adipose tissue preparation and fatty acids analysis*

Samples of adipose tissue were prepared during the first surgery, free of epithelial tissue, and kept frozen in liquid nitrogen until analysis. All analyses were performed in a blinded manner. The procedures for preparation of fatty acids have been reported elsewhere (Chajès *et al.*, 1992). In summary, total lipids of the adipose tissue were extracted, and triglycerides purified on silica gel G thin-layer chromatographic plates. Fatty acids were analysed as methyl esters by gas chromatography on a fused-silica capillary column with a liquid phase of Carbowax 20 M, using an on-column injector and a flame ionisation detector, under operating conditions already described (Martin *et al.*, 1991). Fatty acids were identified and quantified with the use of commercial standards (Nu-Check-Prep, Elisian, MN, USA). All solvents were high-performance liquid chromatography (HPLC) grade, and nitrogen was used at each step. Inter-assay and intra-assay coefficients of variation have been reported (Chajès *et al.*, 1992).

### *Statistical analysis*

Data were analysed using the SAS statistical software (SAS/STAT User's Guide, Version 6, 4th edn, Cary, NC, SAS Institute). Each fatty acid was expressed as a percentage of the total peak area. The associations between fatty acids and clinical characteristics of the patients were measured by Spearman's rank-order correlation coefficients. Log-rank tests (Mantel & Haenszel, 1959) were used to study the prognostic value on metastasis occurrence of conventional prognostic factors and of adipose tissue fatty acids. For each fatty acid, the median content was used as a cut-off value. The metastasis-free curves were estimated by the Kaplan–Meier method (Kaplan & Meier, 1958). The initial time of the study was the initial treatment of the tumour (surgery or preoperative chemotherapy), the end-point date was the date of first metastasis or of last follow-up. All prognostic factors reaching the 10% significance level in the univariate log-rank test were included in the multivariate Cox regression analysis (Cox, 1972).

### Results

### *Patients*

During the observation period (mean 31.2 months; range 8–51 months), 21 patients developed visceral metastases. The prognostic value on the risk of metastases has been studied for conventional factors (Table I). A tumour diameter larger than 50 mm increased the risk by 5.5-fold ($P = 0.0003$). The risk of metastases was 2.6 times greater when the level of oestrogen receptors was below 10 fmol mg$^{-1}$ than when it was above this value.

Initial axillary lymph node involvement was not determined for the 29 patients who received preoperative chemotherapy because of the large size of their tumours. Among the 92 other patients, 44 had axillary lymph node involvement. The risk of metastases did not vary significantly with nodal status or progesterone level.

### *Univariate analysis of breast asipose tissue fatty acids (Table II)*

The $18:3_{n-3}$ level in breast fat was significantly related to the risk of subsequent metastases. The risk was divided by 5 when the level of $18:3_{n-3}$ was above 0.38% compared with when it was lower. Low levels of palmitic acid (16:0) and of docosahexaenoic acid ($22:6_{n-3}$) were also associated with an increased risk of metastasis (Table II).

### *Multivariate analysis*

The $18:3_{n-3}$ level and tumour size were the variables found to increase the global predictive value of the Cox model (Table III). The improvement subsequent to entering other variables was not statistically significant. The risk of metastasis was multiplied by 4.3 when the breast fat $18:3_{n-3}$ level was lower than 0.38% as compared with when it was greater than 0.38%. The risk was multiplied by 4.7 when the diameter of the tumour was larger than 5 cm.

Since the α-linolenic acid level in breast fat seemed to be the first independent predictive factor of prognosis, actuarial probability of metastasis-free survival was plotted in the two groups of patients defined by the $18:3_{n-3}$ level (Figure 1). The relationship between $18:3_{n-3}$ level and prognostic factors was examined. A low level of $18:3_{n-3}$ in adipose breast tissue was associated with positive axillary lymph node status and with vascular invasion, but not with tumour size or mitotic index.

**Table I** Clinical characteristics of breast tumours and risk of metastasis

| *Prognostic factor* | *Patients (n = 121)* | *Patients with metastasis* | *Relative risk of metastasis (95% confidence interval)* | P-*value*[a] |
|---|---|---|---|---|
| Tumour size (mm) | | | | |
| ⩽50 | 103 | 13 | 1.0[b] | |
| >50 | 18 | 8 | 5.5 (2.2–13.8) | 0.0003 |
| Nodal status[c] | | | | |
| Negative | 48 | 7 | 1.0[b] | |
| Positive | 44 | 10 | 1.7 (0.6–4.4) | NS[d] |
| Mitotic index[e] | | | | |
| I or II | 61 | 6 | 1.0[b] | |
| III | 58 | 15 | 2.9 (1.1–7.5) | 0.03 |
| Vascular invasion[f] | | | | |
| Absent | 76 | 9 | 1.0[b] | |
| Present | 41 | 12 | 2.8 (1.2–6.6) | 0.02 |
| Oestrogen receptor (fmol mg$^{-1}$)[f] | | | | |
| ⩾10 | 101 | 15 | 1.0[b] | |
| <10 | 16 | 5 | 2.6 (0.9–7.1) | 0.06 |
| Progesterone receptor (fmol mg$^{-1}$)[f] | | | | |
| >10 | 85 | 13 | 1.0[b] | |
| ⩽10 | 32 | 7 | 1.8 (0.7–4.5) | NS[d] |

[a]Log-rank test. [b]Reference category. [c]Unavailable for 29 patients with preoperative chemotherapy. [d]NS, not significant ($P > 0.10$). [e]Unavailable for two patients. [f]Unavailable for four patients.

**Table II** Univariate analysis on fatty acids in breast adipose tissue

| *Fatty acid (%)*[a] | *Patients (n = 121)* | *Patients with metastasis (n = 21)* | *Relative risk of metastasis (95% confidence interval)* | P-*value*[b] |
|---|---|---|---|---|
| *Saturates* | | | | |
| 16:0 (palmitic acid) | | | | |
| <22.9 | 61 | 14 | 1.0[c] | |
| ⩾22.9 | 60 | 7 | 0.4 (0.1–0.9) | 0.03 |
| 18:0 (stearic acid) | | | | |
| <5.94 | 61 | 11 | 1.0[c] | |
| ⩾5.94 | 60 | 10 | 0.7 (0.3–1.6) | NS[d] |
| *Monounsaturates* | | | | |
| 16:1 (palmitoleic acid) | | | | |
| <3.81 | 61 | 11 | 1.0[c] | |
| ⩾3.81 | 60 | 10 | 0.9 (0.4–2.2) | NS[d] |
| $18:1_{n-9}$ (oleic acid) | | | | |
| <42.05 | 61 | 11 | 1.0[c] | |
| ⩾42.05 | 60 | 10 | 0.9 (0.4–2.1) | NS[d] |
| *Polyunsaturates n-6* | | | | |
| $18:2_{n-6}$ (linoleic acid) | | | | |
| <14.58 | 60 | 9 | 1.0[c] | |
| ⩾14.58 | 61 | 12 | 1.3 (0.5–3.0) | NS[d] |
| $20:4_{n-6}$ (arachidonic acid) | | | | |
| <0.30 | 59 | 9 | 1.0[c] | |
| ⩾0.30 | 62 | 12 | 1.7 (0.7–4.1) | NS[d] |
| $22:4_{n-6}$ (nervonic acid) | | | | |
| <0.14 | 59 | 9 | 1.0[c] | |
| ⩾0.14 | 62 | 12 | 1.7 (0.7–4.1) | NS[d] |
| *Polyunsaturates n-3* | | | | |
| $18:3_{n-3}$ (α-linolenic acid) | | | | |
| <0.38 | 60 | 17 | 1.0[c] | |
| ⩾0.38 | 61 | 4 | 0.2 (0.1–0.6) | 0.004 |
| $22:5_{n-3}$ (docosapentaenoic acid) | | | | |
| <0.20 | 58 | 14 | 1.0[c] | |
| ⩾0.20 | 63 | 7 | 0.5 (0.2–1.2) | NS[d] |
| $22:6_{n-3}$ (docosahexaenoic acid) | | | | |
| <0.17 | 56 | 14 | 1.0[c] | |
| ⩾0.17 | 65 | 7 | 0.4 (0.2–1.0) | 0.06 |

[a]Percentage of total fatty acids. [b]Log-rank test. [c]Reference category. [d]NS, not significant.

**Table III** Multivariate analysis of prognostic factors

| *Prognostic factor* | *Relative risk (95% confidence interval)* | P-*value*[a] |
|---|---|---|
| α-Linolenic acid ($18:3_{n-3}$) (%) | | |
| >0.38 | 1.0[b] | 0.009 |
| ⩽0.38 | 4.3 (1.4–13) | |
| Tumour clinical size (mm) | | |
| ⩽50 | 1.0[b] | 0.001 |
| >50 | 4.7 (1.9–12) | |

Global chi-square: 19.5 with 2 d.f. ($P = 0.0001$). [a]Wald test. [b]Reference category.

**Figure 1** Metastasis-free survival according to the breast adipose tissue level of $18:3_{n-3}$. The threshold for $18:3_{n-3}$ is the median (log-rank value 10.29, $P<0.001$).

### Discussion

We present data relating occurrence of metastases to a decreased level of α-linolenic acid in adipose breast tissue of breast cancer patients. Although local relapses may reflect the biological aggressiveness of the carcinoma in a way similar to the development of distant metastases, these events were not taken into account in the present study because their occurrence is known to be influenced by the local treatment.

The site of sampling that we used was the breast, close to the carcinoma. Metabolic interactions exist between adipose tissue and breast epithelium (Kidwell, 1989; Carroll & Parenteau, 1992), and the tumour may recruit its fatty acids from breast adipocytes. The properties of $18:3_{n-3}$ may make its selective removal plausible. White adipose tissue fatty acids have different rates of mobilisation, which increases with their unsaturation and reduced chain length and is greater for n-3 than for n-6 polyunsaturates (Raclot & Groscolas, 1993). Thus, $18:3_{n-3}$ has a higher relative mobilisation index than other major fatty acids commonly found in human adipose tissue. Like other essential fatty acids, $18:3_{n-3}$ is desaturated and elongated to produce long-chain n-3 polunsaturates. But its metabolism differs in that it is more rapidly oxidised than all other 16- to 22-carbon fatty acids (Cunnane, 1991), and it is always present in very low amounts in storage tissues. Therefore, the possibility exists that the low level of $18:3_{n-3}$ observed in breast cancer patients with poor prognosis could be a consequence of its high rate of mobilisation, or of its preferential oxidation to fulfil specific needs for tumour growth and/or host aggression. However, the lack of correlation observed between the level of $18:3_{n-3}$ in the adipose tissue and the size of the primary tumour or the mitotic index suggests that a low level of $18:3_{n-3}$ in breast fat is not induced by growth of the vicinal, primary tumour, and makes it unlikely that it might be the consequence of distant occult metastatic growth occurring simultaneously in the same patient.

We have previously reported that in breast cancer patients the $18:3_{n-3}$ level in breast adipose tissue is correlated with the $18:3_{n-3}$ level in gluteal adipose tissue (Chajès *et al.*, 1992), suggesting that its level in breast might reflect the body reserves of this fatty acid. A possible cause of depleted $18:3_{n-3}$ in adipose stores is insufficient intake of this fatty acid. In contrast to the numerous reports relating long-chain polyunsaturate intake with the corresponding fatty acid content of adipose tissue, there have been few studies of the association between the intake of $18:3_{n-3}$ and its level in adipose tissue. A weak correlation with dietary intake was reported for this fatty acid in a recent study, but the correlation was much higher in the subpopulation of patients with stable weight (London *et al.*, 1991). This agrees with the observation that this fatty acid is selectively oxidised during weight loss achieved by low-calorie dieting in obese patients (Phinney *et al.*, 1990; Hudgins & Hirsch, 1991). No weight loss had occurred in our population of patients at the time of analysis of their adipose tissue. Therefore breast cancer patients with a low level of $18:3_{n-3}$ in adipose breast tissue may have a reduced dietary intake of $18:3_{n-3}$.

The possibility that dietary fat might alter the outcome of breast cancer has been examined in some epidemiological studies, and conclusive evidence for a role of one individual fatty acid has been precluded by methodological limitations in the measurement of fatty acid intake (Greenwald, 1989; Prentice *et al.*, 1989). Decreased breast cancer incidence and improved survival has been reported to be associated with a high proportion of dietary fat originating from fish (Kaizer *et al.*, 1989) enriched in long-chain n-3 polyunsaturated fatty acids (PUFAs). Among the dietary surveys, one found that the risk of death from breast cancer increased with fat intake (Gregorio *et al.*, 1985), and another did not (Newman *et al.*, 1986). However, the dietary questionnaires used in these studies did not allow qualitative estimation of the type of fat ingested. Recently, associations have been reported between total fat or saturated and polyunsaturated fat intakes evaluated at the time of diagnosis and treatment failure in oestrogen-receptor positive breast cancer (Holm *et al.*, 1993). However, no details were available to distinguish n-6 or n-3 families of polyunsaturates for their possible opposite effects on relapses. In contrast, the Multiple Risk Factor Intervention Trial (MRFIT) reported significant associations between cancer mortality and the ratio of dietary $18:3_{n-3}$ to $18:2_{n-6}$, or the ratio of total n-3 to n-6 PUFAs (Dolecek, 1992). Although the study did not involve breast cancer, it suggests that the composition of dietary fat, and specifically its content of different essential fatty acids, may influence cancer rates and death. In experimental animal models of mammary carcinogenesis, high n-6 PUFA diets stimulate mammary tumour growth and development as well as metastases (Carroll, 1986; Erickson & Hubbard, 1990), while long-chain n-3 PUFA (Adams *et al.*, 1990; Cave, 1991) or $18:3_{n-3}$ enrichment of the diet (Tinsley *et al.*, 1981; Kamano *et al.*, 1989; Fritsche & Johnston, 1990; Hirose *et al.*, 1990) inhibits tumour growth. Therefore, in the rat, the effects of dietary long-chain n-3 PUFAs seem to oppose the stimulation of tumour growth induced by n-6 essential fatty acids (Lands, 1992), and $18:3_{n-3}$ appears also to bear inhibitory properties.

There is presently no direct mechanism relating $18:3_{n-3}$ level in the adipose breast tissue to properties of the tumour cells or to host-dependent factors leading to subsequent development of visceral metastases. In contrast with this situation, other prognostic markers of breast cancer frequently indicate properties specifically related to the invasive behaviour of cancer cells (Koscielny *et al.*, 1989), or properties related to molecular alterations of the tumour cells (i.e. the presence of steroid hormone receptors or growth factor receptors, acquired genetic abnormalities) or properties related to the response of the host to tumour growth. Several markers of breast cancer prognosis derived from the stromal response of the host have recently been described, such as stromelysin 3 secretion by stromal cells (Basset *et al.*, 1990) or the angiogenic response of the host to the tumour growth (Weidner *et al.*, 1992). The fact that a low $18:3_{n-3}$ level was related to positive lymph node status and to the presence of vascular invasion suggests a possible association between this fatty acid and mechanisms of tumour cell invasiveness.

The main cause of death in breast cancer patients is the development of distant metastases. Since a reduced $18:3_{n-3}$ content of breast adipose tissue appears to be the first determinant of their occurrence in our series of patients, dietary supplementation of breast cancer patients in conditions leading to a replenishment of adipose stores of $18:3_{n-3}$ might delay or even prevent their clinical appearance. If $18:3_{n-3}$ is actually involved in the metastatic process, the mechanisms leading to its reduction in the adipose tissue must be understood before any dietary intervention is contemplated.

We thank F. Fetissof for the pathology, O. Le Floch, A. Reynaud-Bougnoux and S. Chapet for updating the follow-up files of patients, C. Hill and C. Lhuillery for their critical reading of the manuscript and G. Durand for his suggestions on the role of $18:3_{n-3}$.

This work was supported in part by grants from the Ligue Nationale Contre le Cancer (Comités du Cher, Charente et Loir-et-Cher). V. Chajès was the recipient of a fellowship from the Association pour la Recherche sur le Cancer.

### References

ADAMS, L.M., TROUT, J.R. & KARMALI, R.A. (1990). Effect of N-3 fatty acids on spontaneous and experimental metastasis of rat mammary tumour 13762. *Br. J. Cancer*, **61**, 290–291.

BASSET, P., BELLOCQ, J.P., WOLF, C., STOLL, I., HUTIN, P., LIMACHER, J.M., PODHAJCER, O.L., CHENARD, M.P., RIO, M.C. & CHAMBON, P. (1990). A novel metalloproteinase gene specifically expressed in stromal cells of breast carcinomas. *Nature*, **348**, 699–704.

BELURY, M.A., LEYTON, J., PATRICK, K.E., GUMBERLAND, A.G., LOCNISKAR, M. & FISHER, S.M. (1991). Modulation of phorbol ester-elicited events in mouse epidermis by dietary n-3 and n-6 fatty acids. *Prostaglandins, Leukotrienes and Essential Fatty Acids*, **44**, 19–26.

BLOOM, H.J.G. & RICHARDSON, W.W. (1957). Histologic grading and prognosis in breast cancer. *Br. J. Cancer*, **11**, 359–377.

BOUGNOUX, P., CHAJÈS, V., LANSON, M., HACÈNE, K., BODY, G., COUET, C. & LE FLOCH, O. (1991). Prognostic significance of tumor phosphatidylcholine stearic acid level in breast carcinoma. *Breast Cancer Res. Treat.*, **20**, 185–194.

BURNS, C.P. & SPECTOR, A.A. (1990). Effects of lipids on cancer therapy. *Nutr. Rev.*, **48**, 233–240.

CALAIS, G., DESCAMPS, P., CHAPET, S., TURGEON, V., REYNAUD-BOUGNOUX, A., LEMARIE, E. FIGNON, A., BODY, G., BOUGNOUX, P., LANSAC, J. & LE FLOCH, O. (1993). Primary chemotherapy and radiosurgical breast-conserving treatment for patients with locally advanced operable breast cancers. *Int. J. Radiat. Oncol. Biol. Phys.*, **26**, 37–42.

CARROLL, K.K. (1986). Experimental studies on dietary fat and cancer in relation to epidemiological data. *Prog. Clin. Biol. Res.*, **222**, 231–348.

CARROLL, K.K. & PARENTEAU, H.I. (1992). A suggested mechanism for effects of diet on mammary cancer. *Nutrition Res.*, **12**, S159–S161.

CAVE, W.T. (1991). Dietary n-3 (ω-3) polyunsaturated fatty acid effects on animal tumorigenesis. *FASEB J.*, **5**, 2160–2166.

CHAJÈS, V., NIYONGABO, T., LANSON, M., FIGNON, A., COUET, C. & BOUGNOUX, P. (1992). Fatty acid composition of breast and iliac adipose tissue in breast cancer patients. *Int. J. Cancer*, **50**, 405–408.

COX, D.R. (1972). Regression models and life tables (with discussion). *J. Roy. Soc. Stat. B*, **34**, 65–77.

CUNNANE, S.C., CHEN, Z.Y., YANG, J., LIEDE, A.C., HAMADEH, M. & CRAWFORD, M.A. (1991). Alpha-linolenic acid in humans: direct functional role or dietary precursor? *Nutrition*, **7**, 437-439.

DOLECEK, T.A. (1992). Epidemiological evidence of relationships between dietary polyunsaturated fatty acids and mortality in the multiple risk factor intervention trial. *Proc. Soc. Exp. Biol. Med.*, **200**, 177-182.

DONNELLY, T.E., BIRT, D.F., SITTLER, R., ANDERSON, C.L., CHOE, M. & JULIUS, A. (1987). Dietary fat regulation of the association of protein kinase C activity with epidermal cell membranes. *Carcinogenesis*, **8**, 1867-1870.

ERICKSON, K.L. & HUBBARD, N.E. (1990). Dietary fat and tumor metastasis. *Nutr. Rev.*, **48**, 6-14.

FRITSCHE, K.L. & JOHNSTON, P.V. (1990). Effect of dietary alpha-linolenic acid on growth, metastasis, fatty acid profile and prostaglandin production of two murine mammary adenocarcinomas. *J. Nutr.*, **120**, 1601-1609.

GREENWALD, P. (1989). Strengths and limitations of methodologic approaches to the study of diet and cancer: summary and future perspectives with emphasis on dietary fat and breast cancer. *Prev. Med.*, **18**, 163-166.

GREGORIO, D.I., EMRICH, L.J., GRAHAM, S., MARSHALL, J.R. & NEMOTO, T. (1985). Dietary fat consumption and survival among women with breast cancer. *J. Natl Cancer Inst.*, **75**, 37-41.

HIROSE, M., MASUDA, A., ITO, N., KAMANO, K. & OKUYAMA, H. (1990). Effects of dietary perilla oil, soybean oil and safflower oil on 7,12-dimethylbenz(a)anthracene (DMBA) and 1,2-dimethylhydrazine (DMH)-induced mammary gland and colon carcinogenesis in female SD rats. *Carcinogenesis*, **11**, 731-735.

HOLM, L.E., NORDEVANG, E., HJALMAR, M.L., LIDBRINK, E., CALLMER, E. & NILSSON, B. (1993). Treatment failure and dietary habits in women with breast cancer. *J. Natl Cancer Inst.*, **85**, 32-36.

HUDGINS, L.C. & HIRSCH, J. (1991). Changes in abdominal and gluteal adipose tissue fatty acid compositions in obese subjects after weight gain and weight loss. *Am. J. Clin. Nutr.*, **53**, 1372-1377.

KAIZER, L., BOYD, N.F., KRIUKOV, V. & TRITCHLER, D. (1989). Fish consumption and breast cancer risk: an ecological study. *Nutr. Cancer*, **12**, 61-68.

KAMANO, K., OKUYAMA, H., KONISHI, R. & NAGASAWA, H. (1989). Effects of a high-linoleate and a high-alpha-linolenate diet on spontaneous mammary tumorigenesis in mice. *Anticancer Res.*, **9**, 1903-1908.

KAPLAN, E.L. & MEIER, P. (1958). Non parmetric estimations from incomplete observations. *J. Am. Stat. Assoc.*, **53**, 457-465.

KIDWELL, W.R. (1989). Differential responsiveness of normal and neoplastic mammary epithelium to unsaturated *vs* saturated fatty acids. In *Carcinogenesis and Dietary Fat*, Abraham, S. (ed.) pp. 417-423. Kluwer Academic Publishers: Boston.

KOSCIELNY, S., LE, M.G. & TUBIANA, M. (1989). The natural history of human breast cancer: the relationship between involvement of axillary lymph nodes and the initiation of distant metastases. *Br. J. Cancer*, **59**, 775-782.

LANDS, W.E.M. (1992). Biochemistry and physiology of n-3 fatty acids. *FASEB J.*, **6**, 2530-2536.

LANSON, M., BOUGNOUX, P., BESSON, P., LANSAC, J., HUBERT, B., COUET, C. & LE FLOCH, O. (1990). N-6 polyunsaturated fatty acids in human breast carcinoma phosphatidylethanolamine and early relapse. *Br. J. Cancer*, **61**, 776-778.

LONDON, S.J., SACKS, F.M., CAESAR, J., STAMPFER, M.J., SIGUEL, E. & WILLETT, W.C. (1991). Fatty acid composition of subcutaneous adipose tissue and diet in postmenopausal US women. *Am. J. Clin. Nutr.*, **54**, 340-345.

MANTEL, N. & HAENSZEL, W. (1959). The statistical aspects of the analysis of data from retrospective studies of a disease. *J. Natl Cancer Inst.*, **22**, 719-748.

MARTIN, J.C., NIYONGABO, T., MOREAU, L., ANTOINE, J.M., LANSON, M., BERGER, C., LAMISSE, F., BOUGNOUX, P. & COUET, C. (1991). Essential fatty acid composition of human colostrum triglycerides: its relationship with adipose tissue composition. *Am. J. Clin. Nutr.*, **54**, 829-835.

NEWMAN, S.C., MILLER, A.B. & HOWE, G.R. (1986). A study of the effects of weight and dietary fat on breast cancer survival time. *Am. J. Epidemiol.*, **123**, 767-774.

PHINNEY, S.D., TANG, A.B., JOHNSON, S.B. & HOLMAN, R.T. (1990). Reduced adipose 18:3ω3 with weight loss by very low calorie dieting. *Lipids*, **25**, 798-806.

PRENTICE, R.L., PEPE, M. & SELF, S.G. (1989). Dietary fat and breast cancer: a quantitative assessment of the epidemiological literature and a discussion of methodological issues. *Cancer Res.*, **49**, 3147-3156.

RACLOT, T. & GROSCOLAS, R. (1993). Differential mobilization of white adipose tissue fatty acids according to chain length, unsaturation and positional isomerism. *J. Lipid Res.*, **34**, 1515-1526.

SCHATZKIN, A., GREENWALD, P., BYAR, D.P. & CLIFFORD, C.K. (1989). The dietary fat breast cancer hypothesis is alive. *JAMA*, **261**, 3284-3287.

SPEIZER, L.A., WATSON, M.J. & BRUNTON, L.L. (1991). Differential effects of omega-3 fish oils on protein kinase activities *in vitro*. *Am. J. Physiol.*, **261**, E109-E114.

TINSLEY, I.J., SCHMITZ, J.A. & PIERCE, D.A. (1981). Influence of dietary fatty acids on the incidence of mammary tumors in the C3H mouse. *Cancer Res.*, **41**, 1460-1465.

WEIDNER, N., FOLKMAN, J., POZZA, F., BEVILACQUA, P., ALLRED, E.N., MOORE, D.H., MELI, S. & GASPARINI, G. (1992). Tumor angiogenesis: a new significant and independent prognostic indicator in early stage breast carcinoma. *J. Natl Cancer Inst.*, **84**, 1875-1887.